# Supplementary material for: The genome of the Paleogene relic tree Bretschneidera sinensis: insights into trade-offs in gene family evolution, demographic history, and adaptive SNPs
Source: DNA Res. 2022 Feb 4;29(1):dsac003. doi: 10.1093/dnares/dsac003 (PMC8825261; doi:10.1093/dnares/dsac003)
Supplement: dsac003_Supplementary_Data [file dsac003_supplementary_data.zip › DNA_research-tracked.docx]

Running title: *Bretschneidera sinensis* genome

**The genome of the Paleogene relic tree *Bretschneidera sinensis*: insights into trade-offs in gene family evolution, demographic history, and adaptive SNPs**

Hai-Lin Liu^1, 2, 3, 4^, AJ Harris^1, 5^, Zheng-Feng Wang^1, 6, 7, 8*^,

Hong-Feng Chen^1, 5*^, Zhi-An Li ^1, 6, 7, 8^, Xiao Wei^9^

^1^ *Guangdong Provincial Key Laboratory of Applied Botany, South China Botanical Garden, Chinese Academy of Sciences, Guangzhou, China*

^2^ *University of Chinese Academy of Sciences, Beijing, China*

^3^ *Environmental Horticulture Research Institute, Guangdong Academy of Agricultural Sciences, Guangzhou, China*

^4^ *Key Lab of Ornamental Plant Germplasm Innovation and Utilization, Guangzhou, China*

^5^ *Key Laboratory of Plant Resources Conservation and Sustainable Utilization, South China Botanical Garden, Chinese Academy of Sciences, Guangzhou, China*

^6^ *Southern Marine Science and Engineering Guangdong Laboratory (Guangzhou), Guangzhou, China*

^7^ *Center of Plant Ecology, Core Botanical Gardens, Chinese Academy of Sciences, Guangzhou, China*

^8^ *Key Laboratory of Vegetation Restoration and Management of Degraded Ecosystems, South China Botanical Garden, Chinese Academy of Sciences, Guangzhou, China*

^9^ *Guangxi Institute of Botany, Chinese Academy of Sciences, Guilin, China*

*To whom correspondence should be addressed. Email: wzf@scib.ac.cn; h.f.chen@scib.ac.cn

Address:

South China Botanical Garden,

Chinese Academy of Sciences,

Guangzhou, Guangdong, 510650

China

**Abstract**

Among relic species, genomic information may provide the key to inferring their long-term survival. Therefore, in this study, we investigated the genome of the Paleogene relic tree species, *Bretschneidera sinensis*, which is a rare endemic species within southeastern Asia. Specifically, we assembled a high-quality genome for *B. sinensis* using PacBio HiFi and Hi-C reads and annotated it with long and short RNA-seq reads. Using the genome, we then detected a trade-off between active and passive disease defenses among the gene families. Gene families involved in salicylic acid (SA) and MAPK signaling pathways expanded as active defense mechanisms against disease, but families involved in terpene synthase activity as passive defenses contracted. When inferring the long evolutionary history of *B. sinensis*, we detected population declines corresponding to historical climate change around the Eocene–Oligocene transition and to climatic fluctuations in the Quaternary. Additionally, based on this genome, we identified 388 SNPs that were likely under selection, and showed diverse functions in growth and stress responses. Among them, we further found 41 climate-associated SNPs. The genome of *B. sinensis* and the SNP dataset will be important resources for understanding extinction/diversification processes using comparative genomics in different lineages.

**Keywords:** Gene family; Genome assembly; Population genetics; Resequencing; SNP

1. **Introduction**

Relic species are surviving members of lineages that were once widespread geographically and/or contained considerable taxonomic diversity that is lacking today. Although many relict species are currently confined to historical refugia and are endangered, they are remarkable for their persistence, while other related lineages or populations have become extinct. Understanding how relic species have survived historical environmental changes, including under recent anthropogenic influence, is fundamental to conservation and restoration studies.^1-7^ Considering many relic lineages contain only a few species or are monotypic,^3^ their genomes can provide species-specific information, such as on gene family evolution, demographic history, and adaptive SNPs, to support long-term survival. With the rapid development of genome sequencing techniques, it is increasingly possible to obtain the whole genomes relatively easily, which benefits the understanding and conservation of relic species.

The relic species, *Bretschneidera sinensis* Hemsl (2*n* = 18) (Figure 1),^8^ is a relic deciduous broad-leaved forest tree that was once a component of the boreotropical flora found throughout the Northern Hemisphere.^9^ However, in modern times, *B. sinensis* is regionally endemic and mainly restricted to elevations of 300-1,700 in remote mountainous areas of southern China at a latitude between 20°N and 30°N with other scattered individuals occurring in northern Vietnam, Thailand, and Myanmar. As with many relic plants, all natural population sizes of *B*. *sinensis* are small,^10^ often comprising 30 or less mature individuals in each population based on field investigations.^11^ Presently, the species is listed as a Category-I endangered species in the “Key List of Protection of Wild Plants in China” and as endangered globally by the International Union for Conservation of Nature (IUCN) Red List.^12,13^ Despite its small populations and concerning conservation status, *B. sinensis* occupies a larger geographic breadth than many relic species, and, therefore, occurs in a relatively wide range of environments. This indicates that this species may have an evolutionary mechanism or adaptive genetic variation that supports its persistence.

Since *B*. *sinensis* was first discovered at the end of the 19th century, its taxonomic status has been controversial.^10,14,15^ Within various taxonomic treatments, it has been classified into different families and orders, including Brassicales and Sapindales. However, classification systems broadly agree that the *Bretschneidera* genus should be regarded as monotypic because no close living relatives of the species are known to exist. Moreover, treatments also agree that *B. sinensis* belongs within the malvids lineage, which includes both Brassicales and Sapindales.^16^ The phylogeny using the complete chloroplast genome sequence of *B. sinensis* and other species of malvids further indicated that *B. sinensis* is genetically closest to *Carica* *papaya*.^17^

In addition to taxonomy, other studies on *B. sinensis* have primarily focused on its conservation, especially to identify the factors that possibly underlie its rarity and endangered status, such as seed germination,^18^ plant growth rates,^19,20^ water and light utilization,^21,22^ leaf and root anatomy,^23-25^ reproductive strategies,^26,27^ and genetic diversity.^10,11^ Results from these studies showed that, in the lab, the seed germination rate of *B. sinensis* was about 70%, but can be increased to 80% with gibberellic acid (GA3) treatment.^18^ However, the seed germination rate dramatically decreases to ~ 15% in the field.^20^ With respect to growth rate, *B. sinensis* is a slow- to medium-growing plant exhibiting a curvilinear pattern of height gain; increasing in rate from the seeding to juvenile stage (~0.26 m/year) but decreasing in the second and third years (~0.18 m/year).^19,20^ The growth rate gradually increases again beyond the fourth year, but at only ~0.40 m/year, and remains lower than many other local tree species.^19^ During growth, *B. sinensis* requires shade for the seedling stage and full sun at maturity.^25,28,29^ It prefers a moist climate and is highly sensitive to drought at all stages of growth,^20-22,29^ including during seed dormancy and germination.^30^

The reproductive strategy of *B. sinensis* includes typical insect pollination of extremely attractive, strongly zygomorphic white flowers containing pink or reddish veins, but its pollen grains are relatively large and not sticky and are, thus, not easily carried by insects.^26,27^ Moreover, *B. sinensis* has a stigma that excretes a relatively small amount of fluid and a small receptacle. These factors contribute to low pollination and fertilization rates in *B. sinensis* and therefore low fecundity. However, this species has an outcrossing breeding system through which it maintains genetic diversity,^11^ leading to cautious optimism that conservation of the species is feasible. Nevertheless, taken together, the growth and reproduction strategies of *B. sinensis* may lead to a lower competitive ability compared to other tree species within plant communities where it occurs, thus explaining its rarity.^26,27,31^

Anatomical studies of leaves and roots of *B. sinensis* have also shed light on the potential causes of its rarity^23-25^ because these two organ systems are responsible for light, water, and nutrition absorption and are, thus, tied to plant growth. In particular, studies of leaf anatomy revealed that seedlings of *B. sinensis* have leaves with a higher trichome density, the presence of flower-like papillae, and thinner cuticles compared to leaves of mature individuals.^24,25^ The trichomes, papillae, and cuticles are useful in preventing water loss from the leaf, and papillae may also function in increasing the leaf surface area, consequently enhancing the absorbance of diffuse light within the forest understory during the seedling stage.^25^ Thus, these structures appear to represent highly adaptive strategies for *B. sinensis*. However, studies of root anatomy revealed that the roots of *B. sinensis* lack root hairs.^23^ Root hairs are critical for the absorption of water and soil nutrients. Therefore, the lack of root hairs in this species may yield slower growth rates and decreased competitive ability.

Although prior studies have elucidated many aspects of *B. sinensis*, no studies have investigated its gene family evolution, demographic history and potential adaptive genetic variation, which could elucidate the genomic and/or environmental mechanisms of its long-term persistence and aid its restoration. Therefore, to illustrate these within *B. sinensis*, we generated and annotated a high-quality reference genome for the species, resequenced geographically representative populations, and mapped the resequencing data to the assembled genome to obtain single nucleotide polymorphisms (SNPs). We used PacBio high-fidelity (HiFi) and Hi-C reads for reference genome assembly, transcripts from both PacBio isoform and Illumina **RNA** sequencing for genome annotation, and Illumina short reads for resequencing. We believe that the *B. sinensis* genome and the newly identified SNPs will not only benefit conservation efforts in this species but also provide a pipeline that can be applied to elucidate the genomic basis for the modern-day relic status of other, similar species.

1. **Materials and methods**
   1. **Library construction and sequencing**

For genome assembly, we sampled fresh leaves from one *B. sinensis* individual planted in the South Botanical Garden, China (SCBG, Guangzhou, China). From this leaf material, we constructed a total of three genomic libraries and two transcriptomic libraries for five different next-generation sequencing approaches. These comprised PacBio long-read libraries for circular consensus sequencing of DNA (PacBio HiFi) and full-length RNA transcript sequencing (Iso-Seq), Illumina short-read libraries for whole genome sequencing (WGS) and RNA-Seq, and a high-throughput chromosome conformation capture (Hi-C) library for genome scaffolding. For DNA sequencing using Illumina and PacBio, we extracted genomic DNA using a modified cetyltrimethylammonium bromide (CTAB) protocol.^32^ For the Hi-C library, we extracted genomic DNA following cross-linkage with formaldehyde. Additionally, we extracted the total RNA from *B. sinensis* leaves and then synthesized them to cDNA for the Illumina RNA-Seq and Iso-Seq libraries. All library preparations and sequencing were carried out by the Annoroad Gene Technology Company (Beijing, China). Illumina short read sequencings were performed using an Illumina HiSeq X Ten platform with 150 bp paired end reads (PE-150 bp) and long read sequencing with the PacBio Sequel II platform. For resequencing of *B. sinensis* individuals, we applied Illumina HiSeq X Ten with PE-150 bp to 154 individuals from 13 populations (Table 1, Figure 2A). Each sample yielded about 20 Gb of data.

After all sequencing was completed, we performed genome assembly, annotation, mapping, and SNP calling. For all data analyses, we ran programs under default settings except where otherwise indicated below.

- 1. **Genome assembly and evaluation**

Prior to genome assembly, we removed reads with **base** quality scores **less than 30 and lengths shorter than 80 bp from the raw** Illumina WGS and Hi-C datasets using **Sickle v1.33** (https://github.com/najoshi/sickle). We further corrected the Illumina WGS reads in RECKONER v1.1.0^33^ and applied the result for genome size estimation of *B*. *sinensis* in KmerGenie v1.7044^34^ under the parameters “--diploid -*k* 141”. We also employed these reads to estimate the level of heterozygosity in the *B*. *sinensis* genome with GenomeScope 2.0^35^ under the *k*-mer size of 21 and maximum *k*-mer coverage of 10,000. We processed the raw PacBio HiFi reads using CCS algorithm v4.2.0 (<https://github.com/PacificBiosciences/ccs/releases/tag/v4.2.0>.) to obtain consensus reads, which we assembled in Hifiasm v0.11 (<https://github.com/chhylp123/hifiasm>). After assembly, we used both Pseudohaploid (<https://github.com/schatzlab/pseudohaploid>) and Purge_Dups v1.2.5^36^ to examine and remove duplications in the genome. We identified areas of potential mis-assembly and determined breaks in the contigs using Scaffhic v1.1 (<https://github.com/wtsi-hpag/scaffHiC>) according to the Illumina Hi-C reads. Thereafter, we applied the Juicer pipeline v1.6^37^ and 3d-dna v180114^38^ to perform scaffolding, and we visualized the results in Juicebox v1.11.08^39^ and manually corrected the errors. We further refined the scaffolds in MisjoinDetect (<https://github.com/caixu0518/MisjoinDetect>) before re-running the Juicer pipeline, 3d-dna, and juicebox. For the final scaffolded genome, we used LR_Gapcloser^40^ to close gaps, and we performed a final check for possible duplications by re-running Purge_Dups.

To evaluate the quality of assembly, we used the eudicots_odb10 database within Benchmarking Universal Single-Copy Orthologs (BUSCO) v4.0.6.^41^ We also assessed quality based on the Illumina WGS reads in SQUAT^42^ through read mapping quality analytics with the parameter “-sample-size 200,000,000”. SQUAT uses two different alignment algorithms, BWA-MEM and BWA-backtrack, to assess mapping quality and report the percentages of uniquely mapped, multiply mapped, and unmapped reads. For uniquely mapped reads, SQUAT further classifies them into additional categories, such as those that are perfectly matched, those containing substitutions, those with mismatches at the ends (i.e., clips), and others.

**2.3. Repetitive sequence annotation**

To identify repeat sequences within the *B*. *sinensis* genome, we used EDTA v1.8.3^43^ and RED v2.0^44^ and combined the results using the “merge” command in bedtools v2.29.2.^45^ Based on the merged outcome, we masked repeat sequences using the “maskfasta” command in bedtools.

**2.4. Gene prediction and annotation**

We performed gene prediction in LoReAn v2,^46^ an automated pipeline designed for the annotation of eukaryotic genomes. In addition to conducting ab initio gene prediction in LoReAn, we used the program for gene prediction based on RNA- and Iso-seq reads and protein sequences (Supplementary Table S1). The protein sequences were used for homology-based gene prediction. We chose them from the generally related species in the order of Brassicales and Sapindales in which *B*. *sinensis* was supposed to belong (see introduction), but more species in the former were selected considering the phylogeny shown in Huang et al.^17^ For the Iso-seq reads, we first processed them in IsoSeq v3 (<https://github.com/ben-lerch/IsoSeq-3.0>) to obtain the full-length transcripts. After LoReAn prediction, we used the results as input for the funannotate pipeline v1.8.2 (<https://github.com/nextgenusfs/funannotate>) to acquire final integrated and consensus gene sets using the command of “funannotate predict.” During funannotate prediction, we applied the parameters “-max_intronlen 100,000 -busco_db embryophyta -organism other”.

After gene prediction, we further used the funannotate pipeline for gene functional annotation with the command “funannotate annotate.” The annotation databases used included dbCAN v8.0,^47^ eggNOG v5.0,^48^ which includes GO (Gene Ontology)^49,50^ and KEGG,^51^ InterPro v79,^52^ MEROPS v12.2,^53^ Pfam v32.0,^54^ and UniProt v2020_02.^55^ We also performed secretome prediction in SignalP v4.1.^56^

**2.5. Orthologous gene group identification and analysis**

We used OrthoFinder v2.4.0^57,58^ to identify orthologous gene groups representing gene families within *B*. *sinensis* as well as in 11 other species representing malvids and species from large, related clades based on publicly available data (Supplementary Table S2). After the identification of orthologous groups, we allowed OrthoFinder to automatically select single copy orthologs to generate the species trees. We used the species tree to obtain a dated phylogeny in TreePL,^59,60^ in which we set nine time calibration points for species pairs using their divergence time data (million years ago, MYA) from the Timetree database (http://timetree.org/) (Supplementary Table S3).

Based on the dated phylogeny, we estimated the expansions and contractions of orthologous gene families in CAFE v5.^61^ Prior to performing analyses in CAFE, we followed the users’ manual and removed gene families with more than 200 genes. For significantly expanded and contracted gene families, we followed up by conducting enrichment analysis according to the GO and KEGG databases using Tbtools v1.068.^62^ For significantly enriched GO terms, we visualized their inter-relationships in the Direct Acyclic Graph (DAG) with agriGO v2.0^63^ and generated a treemap with REVIGO.^64^

**2.6. Genome comparison**

We also compared the functional annotations of genes among the genomes of *B. sinensis* and eight additional sampled species representing Sapindales, Malvales and Brassicales that comprised a clade with *B. sinensis* in phylogenetic analyses (see Results). To accomplish this, we used the “funannotate compare” command within the funannotate pipeline. This command applies Fisher’s exact test with a Benjamini-Hochberg correction^65^ to conduct enrichment analysis of GO terms. However, because the gene feature file (.gff3) of *Brassica rapa* downloaded from http://brassicadb.org, could not be parsed by the “funannotate compare” command, we annotated its genome following the same procedure carried out on our assembled genome of *B*. *sinensis* and used short- (SRR12214240-SRR12214243, SRR12214246, SRR12214247, SRR12214250, SRR12214251) and long-read RNA-seq data (SRR10259626-SRR10259628) from GenBank for RNA-seq based prediction.

**2.7. Gene duplications and syntenic block identification**

We used the DupGen_finder pipeline^66^ to examine gene duplications in *B*. *sinensis*. In addition to potential whole genome duplications (WGDs), DupGen_finder also identifies tandem duplications (TDs; separated by five or fewer genes), proximal duplications (PDs; separated by 10 or fewer genes), transposed duplications (TRDs; duplications mediated by transposable elements), and dispersed duplications (DSDs; random and non-neighboring duplications) of genes (see Reference 66 for detailed definitions of all types of duplications). All types of duplications were determined based on paired genes within species using an all-versus-all BLASTP search against *Hevea brasiliensis*, which we regarded as an outgroup. We also identified areas of homologous gene regions among chromosomes of *B. sinensis* using MCScan (Python version)^67^ implemented in the jcvi package^68^ and visualized them in Shinycircos.^69^

**2.8. Demographic history analysis**

Based on the assembled genome and our Illumina WGS reads, we inferred the demographic history of *B*. *sinensis* using MSMC2 v2.1.3.^70^ This method of inference requires the mutation rate and generation time of species as input in addition to the genome. Because the mutation rate in *B*. *sinensis* is not available, we used the “evolutionary rate”, which has been shown to have little difference from the mutation rate,^71^ as a surrogate. To determine that, we first aligned the single copy orthologous genes shared among *B. sinensis* and *Carica papaya* (i.e., its sister based on our reconstructed phylogeny; see Results) and outgroup species *Gossypium austral*. We then performed Tajima’s relative rate test to examine if these genes were in the equality of evolutionary rate using the Relative-Rate-Test (https://github.com/lyy005/Relative-Rate-Test). The genes in the equality were selected and concatenated these into a single matrix, and removed gaps. Using this matrix, we calculated the proportion of different loci (*D*) between the sequences of *B. sinensis* and *C. papaya* and used this to estimate substitutions (*k*) according to the formula of *k* = (–3/4) ln (1–4*D*/3) following the Jukes and Cantor model.^72^ Thereafter, we inferred the mutation rate as *k/2t*, where *t* is the divergence time between *B. sinensis* and *C. papaya* from our dated phylogeny. We estimated the generation time of *B. sinensis* to be 20 years based on our field observations of when mature trees become reproductive. Moreover, in the demographic inference, we masked repeated sequences following the guidance of Patil et al.^73^

- 1. **SNP calling and genetic structure**

Using the assembled genome of *B. sinensis* generated in this study as a reference, we applied dDocent v2.7.6,^74^ a bash pipeline, to call the SNPs in 154 individuals of the species. Within this pipeline, we used sickle v1.33 (https://github.com/najoshi/sickle) to trim the paired-end reads of each individual by removing reads with lengths of less than 80 bp **and base** quality values **lower than 30. After SNP calling, we applied the software implemented within** dDocent to filter out SNPs of low quality, those deviating from Hardy–Weinberg equilibrium (HWE), and indels. We further filtered SNPs with Plink v1.9^75^ to exclude SNPs that showed high linkage disequilibrium (LD) based on a threshold value of 0.2 for the squared correlation (*R^2^*) between all SNP pairs.

Using the detected high-quality SNPs, we examined the genetic structure of *B. sinensis* via principal component analysis (PCA) and ADMIXTURE.^76^ We performed the PCA analysis in SNPRelate v1.24.0^77^ and conducted ADMIXTURE in the AdmixPipe v2.0.2 pipeline^78^. Within AdmixPipe, we performed 20 replicates for each possible genetic grouping (*K*) from 1 to 10, and we determined the best value of *K* according to cross-validation (CV) error and change of likelihood (Δ*K*).^79^ For the best value of *K*, we applied CLUMPAK v1.1^80^ to estimate the mean membership coefficient using 20 replicates per individual.

**2.10. Genome-wide scans for local adaptation**

We used two approaches to detect SNPs under selection: PCAdapt v4.3.3^81,82^ and BayPass v2.2^83^. PCAdapt indicated that two principal components were suitable for regressing SNPs, and within this, we implemented a *q*-value (i.e., adjusted *p*) < 0.01 as the threshold for determining outliers. For BayPass, we set the default parameters to estimate the XtX statistic^84^, which is akin to *Fst* but accounts for the variance–covariance structure. Thereafter, we simulated pseudo-observed datasets (100,000 SNPs) to provide a calibrated threshold (99%) so that we could identify SNPs putatively under selection, i.e., adaptive SNPs.

For adaptive SNPs, to detect their association with environments, we reran BayPass by only using adaptive SNPs but combining climate variables. We obtained seven climate variables (Table 1) from 1982 to 2020 from https://disc.gsfc.nasa.gov ^85^ for the specific coordinates of each population in the form of rasters at 10 km spatial resolution. Before input into BayPass, we averaged each climate variable across 39 years. After performing BayPass, we used a Redundancy Analysis (RDA) implemented in the vegan package^86^ to visualize the association results.

Finally, for both adaptive and non-adaptive SNPs, we tested their isolation-by-distance (IBD) using IBD v1.5.2^87^ by regression of pairwise genetic differentiation (*Fst*) with their geographic distance. We calculated pairwise *Fst* between populations using Pixy v1.2.5.beta1.^88^

1. **Results and discussion**
   1. **Genome sequencing and assembly**

The five sequenced libraries yielded ~22 Gb of PacBio HiFi reads after performing consensus calling, ~127 Gb of Illumina WGS reads, ~105 Gb of Illumina Hi-C reads, ~22 Gb of Illumina RNA-seq reads, and ~300 Mb of PacBio Iso-seq reads after performing full-length transcript identification.

The genome size of *B. sinensis* estimated in Kmergenie was 1,134,469,815 bp, and the genome heterozygosty rate was 0.71%. The assembled genomes in Hifiasm consisted of 1,222,797,721 bp, comprising 1,416 contigs and a contig N50 of 24,630,967 bp (Table 2). Among these contigs, 137 remained after processing in Pseudohaploid and Purge_Dups to reduce duplications. The remaining contigs were corrected and scaffolded using the Hi-C reads, and these yielded a final genome that was 1,170,884,685 bp with 1,120,988,409 bp (95.74%) of sequences assembled into nine chromosomes (Figure 3A) and a scaffold N50 value of 137,464,959 bp. In the final assembled genome, the GC content was 35.80%, and the chromosomes ranged in size from 152,689,475 bp (Chromosome 1) to 87,593,448 bp (Chromosome 9).

- 1. **Completeness of the genome and quality evaluation**

Based on BUSCO, we found that 2,270 (97.6%) of the 2,326 expected genes within the core eudicotyledons were captured, including 1,596 complete single-copy genes (68.6%) and 764 complete, duplicated genes (29.0%). There were an additional 17 genes (0.7%) that were fragmented and 39 (1.7%) appeared to be missing. SQUAT revealed that only 1% of reads were poorly mapped, and the percentages of different qualities and completeness of mapped reads are shown in Supplementary Table S4. Moreover, our simple assessment of genome integrity by mapping all Illumina WGS reads to the assembled genome using BWA-MEM indicated that 99.77% of reads were properly mapped.

- 1. **Repeat annotation**

EDTA and RED revealed that 60.47% and 58.38% of the genome represented repetitive regions, respectively. According to EDTA, there were more long terminal repeats (LTRs) than other kinds of repetitive sequences, accounting for 47.62% (557,597,939 bp) of the genome (Supplementary Table S5). The largest proportions of LTRs were Gypsy-like (285,140,648 bp; 24.35%) and Copia-like (185,533,829 bp; 15.85%). LTRs were followed in abundance by terminal inverted repeats (TIRs) comprising 10.21% (119,567,301 bp) of the genome. By combining the results of EDTA and RED, we determined that 788,029,873 bp (67.30%) of the assembled genome consisted of repetitive components and thus was subsequently masked. The density of repeat sequences in the genome is shown in Figure 3B.

Compared to the genome size of *C. papaya*, which was 372 Mbp,^89^ the size of *B. sinensis* was almost three times larger. The two genomes contained a similar GC content, with *C. papaya* at 35.3% and *B. sinensis* at 35.80%. The *B. sinensis* genome contained a higher proportion of repetitive sequences than the *C. papaya* genome which had 51.9% repetitive sequences. However, after excluding repetitive sequences in *B. sinensis*, the remaining sequence size of 382,854,812 bp of *B. sinensis* was still larger than the *C. papaya* genome. Therefore, the large genome size of *B. sinensis* is not only caused by repetitive sequence expansion. Both genomes contained the highest proportion of Gypsy-like repetitive elements in their own genome, and the proportion (27.8%) of this element in *C. papaya* was higher than that in *B. sinensis*. However, considering 10.21% TIR elements in *B. sinensis*, the proportion of these repetitive elements in *C. papaya* was very low, with less than 1% of the genome. TIRs play important roles in the transposition of chromosomal fragments throughout the genome,^90^ altering gene expression and generating genetic diversity.^91^ Therefore, how TIRs influence the genetic variation and subsequently associate with the *B. sinensis* long-term adaptation needs further study in the future.

- 1. **Gene prediction and annotation**

With LoReAn, we identified 42,761 genes within the *B. sinensis* genome. After integrating them via the funannotate pipeline, we inferred a total of 48,870 genes encoding 53,020 proteins. Of these protein-coding genes, 44,685 (84.28%) were annotated to at least one database based on functional annotation (Supplementary Table S6).

- 1. **Orthologous gene groups identification and analysis**

A total of 29,808 putative gene families were identified in OrthoFinder representing 488,061 protein-encoding genes across all 13 analyzed species. For 53,020 protein-encoding genes in *B. sinensis*, 47,025 (88.69%) of them were assigned to 17,270 (57.93 %) gene families and 1,525 gene families containing 7,508 genes were specific to *B. sinensis*. The resulting species tree using OrthoFinder resolved *B. sinensis* within Brassicales and showed that it was a sister to *Carica papaya* (Figure 4). The divergence time between *B. sinensis* and *C. papaya* was estimated in TreePL to be 69.8 MYA.

Ultimately, we performed analyses in CAFE on a total of 13,903 gene families after removing six that showed > 200 copies in at least one species and after CAFE removed 15,899 due to their lack of occurrence on the phylogenetic root. Among the 13,903 gene families, 5,876 families exhibited expansions, and 2,327 families exhibited contractions in *B*. *sinensis* (Figure 4). Moreover, 11 families from the expanded families exhibited significant (*P* < 0.05) expansions, while eight families from the contracted families underwent significant contractions. The significantly expanded and contracted families contained 219 and 17 protein-encoding genes, respectively.

Biological processes of the significantly expanded gene families based on GO enrichment analysis (Supplementary Table S7) could be roughly grouped into four clusters corresponding to resistance to disease, adaption to shade, protein phosphorylation, and signaling (Figure 5, Supplementary figures 1 and 2). According to DAG (Supplementary figure 2), the cluster related to resistance to disease converged to the genes related to positive regulation of defense response to virus by host (GO:0002230), defense response to fungus (GO:0050832), defense response, incompatible interaction (GO:0009814), response to molecule of fungal origin (GO:0002238), plant-type hypersensitive response (GO:0009626), and cellular response to salicylic acid (SA) stimulus (GO:0071446). The lowest-level GO term related to adaptation to shade was response to absence of light (GO:0009646), while protein phosphorylation itself (GO:0006468) was the lowest-level GO term related to that process, and signaling (GO:0023052) was alone in its cluster. In the Molecular Function GO category, the most abundant lowest-level GO terms representing expanded gene families were protein serine/threonine kinase activity (GO:0004674) and calmodulin binding (GO:0005516) (Supplementary figure 3). The significantly contracted genes were all within the Biological Process category and were mostly related to responses to biotic stimuli (GO:0009607, GO:0043207) and interspecies interactions between organisms (GO:0044419) (Supplementary Table S8).

The KEGG enrichment analysis revealed that significantly expanded gene families mainly had functions related to environmental adaptation, plant–pathogen interaction, the MAPK signaling pathway, signal transduction, and protein kinases (Supplementary Table S9). The analyses of KEGG enrichment for the significantly contracted gene families indicated that these genes were especially related to terpenoid biosynthesis, including monoterpenoid biosynthesis, terpenoid backbone biosynthesis, and metabolism of terpenoids and polyketides (Supplementary Table S10).

The above results indicated that the most significantly expanded genes were largely involved in plant defenses and, in particular, linked to disease resistance. Among the expanded gene families, we found that the lowest levels of the GO hierarchy were those pertaining directly to viral and fungal resistance (GO:0002230, GO:0050832, and GO:0002238), as well as hypersensitive responses (GO:0009626), cellular response to SA stimulus (GO:0071446), calmodulin binding (GO:0005516), and protein serine/threonine kinase activity (GO:0004674), all of which are closely associated with disease resistance in plants.^92-98^ Notably, calmodulin and SA are known to be critical within the signaling response pathways involved in plant disease resistance; specifically, calmodulin-binding proteins can affect the biosynthesis of signaling of hormones, such as SA.^96^ Additionally, serine/threonine-protein kinases are receptor-like kinases that typically contain a leucine-rich repeat structure that is used to detect biological pathogens.^92,99^ Moreover, plant hypersensitive responses are effective in defenses against disease but come at the cost of a reduction in biomass accumulation because they cause cell death.^100,101^

Our KEGG enrichment analysis largely agrees with the GO enrichment results and suggests that genes related to disease resistance are expanded within *B. sinensis*. Among them, the MAPK signaling pathway has been demonstrated to actuate responses and resistance to plant disease.^102-104^ MAPK, along with SA, is a core mediator for the hypersensitive response and subsequent cell death.^103,105^

**3.6. Genome comparison**

Genes that were over-represented in *B. sinensis* compared to the other sampled species were mainly related to RNA–DNA hybrid ribonuclease activity (GO:0004523) (Supplementary Table S11, Supplementary figure 4), while under-represented genes were primarily associated with terpene synthase (TPS) activity (GO:0010333), zinc ion binding (GO:0008270), and ADP binding (GO:0043531) (Supplementary Table S11, Supplementary figure 5). Because the genes representing TPS activity (GO:0010333) in *B. sinensis* were significantly under-represented, we detected genes across species bearing TPS domains (Pfam IDs PF03936 and PF01397)^106^ and observed that *B. sinensis* displayed the smallest number of TPS genes (Figure 4). The sister to *B. sinensis*, *C. papaya*, also possessed a small number of TPS genes in comparison to other species.

Terpenes are important natural products with a wide range of applications in plants,^106-108^ but they most often serve as continuously available, passive, toxic defenses against biological enemies^108^. The low number of TPS genes in *B. sinensis* is unexpected because plants generally exhibit high numbers of TPS genes.^106^ Among 44 plant species summarized by Jiang et al.,^106^ only *Physcomitrella patens*, *Marchantia polymorpha*, and *Zostera marina*, which comprise a moss, liverwort, and flowering sea grass, respectively, have smaller numbers of TPS genes than *B. sinensis*. Within *B. sinensis*, the reduced number of TPS genes could represent a trade-off between passive and active defense, where the utilization of SA and MAPK, which are enriched in the species, cause cell death and represent an active approach.

- 1. **Gene duplications and syntenic block identification**

The DupGen_Finder pipeline revealed 17,798 gene pairs in *B. sinensis* resulting from WGD, 2,949 gene pairs from TD, 834 gene pairs from PD, 5,710 gene pairs from TRD, and 8,250 gene pairs from DSD. MCScan revealed 421 syntenic blocks containing 23,017 genes and 23,306 gene pairs. The largest syntenic block size was 30,550,842 bp, and the smallest was 174,612 bp (Figure 3B).

The analysis in BUSCO also showed a relatively high level of gene duplication (29.0%). High levels of duplication in BUSCO analyses may be produced by WGD events or represent mis-assembly due to heterozygous contigs unpurged in the genome.^109^ However, we detected no heterozygous haplotigs in the Purge_Dups analysis, and DupGen_Finder indicated that the majority of the duplicated genes are attributed to WGD. Thus, WGD seems to be the most plausible explanation for the high levels of detected duplication.

In contrast to *B. sinensis*, we found that only 0.8% of genes from the BUSCO database were duplicated within the genome of *C. papaya*, which is resolved as a sister to *B. sinensis* in our reconstructed phylogeny. The total BUSCO for the *C. papaya* genome was 80.7%, suggesting that incompleteness might also contribute to the lower duplication rate. To further verify that the observed rate of duplication in *B. sinensis* was not an artifact of sequencing, we sampled and sequenced an additional individual of the species using PacBio and Illumina DNA sequencing, as described above with assembly via the pb-assembly pipeline v0.06 (<https://github.com/PacificBiosciences/pb-assembly>) and Flye v2.8-b1674^110,111^. The new genome comprised approximately 73 Gb of PacBio long reads and 145 Gb of Illumina short reads. After removing duplications using Pseudohaploid and Purge_Dups (see Methods), analyses in BUSCO for the pb-assembly revealed 73.7% completeness for single-copy genes and 23.4% for duplicated genes. Similarly, the Flye assembly showed 76.1% completeness of single-copy genes and 15.2% completeness of duplicated genes. Although the Flye assembly had relatively low gene duplication, the genome was relatively complete (91.3% vs. 97.1% of pb-assembly). Thus, these results suggest that our detection of duplication in our original result was not artificial and represented true WGD events.

- 1. **Demographic history**

We identified a total of 2,311 orthologous genes shared among *B. sinensis*, *C. papaya* and *G. australe*. After performing a Relative-Rate-Test, 856 genes remained. By concatenating them, the aligned length of the sequence was 761,782 bp. We found a total of 130,040 SNPs between *B. sinensis* and *C. papaya*, and we estimated the mutation rate to be 1.39 × 10^-9^. Therefore, to determine the demographic history of *B. sinensis*, we applied this rate plus the estimated generation time of 20 years within MSMC2. This approach revealed substantial demographic fluctuations in the species over time (Figure 6). In particular, we observed an initial clear population size decline from ca. 40 MYA to 20 MYA. Thereafter, the population size increased slightly, but declined again around 3 MYA. After another slight recovery, it declined dramatically around 0.5 MYA to the present rare state.

The first decline happened around 40 MYA, coinciding with a major, global climatic cooling trend around the Eocene-Oligocene transition,^112-116^ and the second major decline happened around 0.5 MYA, corresponding to the Naynayxungla glaciation (0.5–0.72 MYA), one of the most extensive glaciation events during the Quaternary Period^117^. Similar two-step declines in population size have also been detected in other Paleogene relic species, such as *Ginkgo biloba*,^4^ which shows distribution overlap with *B. sinensis* in China. The timing of population declines in *G. biloba* are not fully consistent with those we found for *B. sinensis*, and this could be due to differences in analytical methods,^73,118^ especially that repetitive sequences were not removed in the analysis of *G*. *biloba*.

- 1. **SNP calling and genetic structure analysis**

We obtained 1,284,251 SNPs from 154 *B. sinensis* individuals. After removing 388 SNPs putatively under selection (see below), 1,283,863 remained for genetic structure analyses. Based on these SNPs, a PCA analysis revealed that the first principal component divided 154 *B. sinensis* samples into western populations (JX, LS, LP, ZJJ, ES, and PB) and eastern ones (WZS, HZ, CY, NP, LQ, JN, and TB) (Figure 2B). The second principal component further separated the TB (Taiwan) population from the other six populations among the eastern groups. In the ADMIXTURE analysis, the CV errors decreased constantly from *K* = 1 to *K* = 10, but the main decrease occurred at *K* = 2 (Supplementary figure S6A). The Δ*K* analysis also indicated that the optimal *K* value was 2 (Supplementary figure S6B). When *K* = 2, the 154 samples were also divided into western and eastern groups according to their geographic distribution (Figure 2C), which agrees with the first principal component in the PCA analysis.

Our results are consistent with a previous phylogeographic study of *B. sinensis* based on three chloroplast DNA fragments.^10^ That study proposed that the western group of populations occurs in an area (i.e., the Yungui Plateau) that was less influenced by Neogene and Quaternary glaciations than other geographic areas of China, while the eastern group may have survived in one or more refugia.^10^ However, the prior study did not include the WZS population from Hainan because it was only discovered recently. Here, we included WZS and found that it was within the eastern group. Although both the WZS and TB populations were separated from mainland China by the Qiongzhou and Taiwan straits, respectively, the TB population showed higher divergence from the other eastern populations than the WZS population based on the second principal component of our PCA analysis (Figure 2B). The timing of the formation of the Qiongzhou and Taiwan straits is still uncertain.^119-125^ Nevertheless, our results indicate that the Taiwan Strait might have formed earlier than the Qiongzhou Strait, leading to longer isolation of the TB population and hence greater divergence.

- 1. **. Genome-wide scans for local adaptation**

PCAdapt identified 259,076 SNPs putatively under selection, while BayPass identified 7,182, and of these, 388 SNPs were identified according to both methods. For the shared SNPs, 94 were in exonic regions of 59 genes, and 22 were in the **three prime** untranslated regions (3’-UTR) of 11 genes (Supplementary Table S12). These genes were presumably related to diverse functions such as involvement in the growth of *B. sinensis* (e.g., *PLIM2B_1*, *JHS1*, and *MES17* genes) and stress response (e.g., *VTE5*, *RH1_3*, and *CYCL11_1*genes) based on their annotations established in this study. These genes have been characterized in *Arabidopsis thaliana* and other model species, in which *PLIM2B* plays a crucial role in actin configuration during pollen germination and tube growth,^126,127^ while *JHS1* plays an important role in DNA replication and damage repair, meristem maintenance, and development.^128^ *MES17* is a plant hormone-related gene and efficiently and specifically hydrolyzes MeIAA (Methyl Indole-3-Acetic Acid) to IAA,^129^ while IAA is known to be important in both root and shoot development in plants.^130,131^ Among the genes related to stress response, *VTE5* encodes phytol kinase and is involved in tocopherol production, and in *A. thaliana*, it is related to resistance to root-knot nematodes,^132^ as well as to high-light and high-temperature stress tolerance in tomato.^133^ *RH1* is in the DEAD-box-containing RNA helicase family, which modulates the secondary and [tertiary structure](https://www.sciencedirect.com/topics/biochemistry-genetics-and-molecular-biology/tertiary-structure) of RNA.^134^ Previous studies indicated that this gene family was involved in many stress responses, such as to disease, drought, salinity, cold, and oxidation.^135,136^ *CYCL11_1* encodes a cognate cyclin for cyclin-dependent kinase G1 and G2 and modulates flowering time in response to temperature.^137-139^ The fact that all of these stress response and growth-related genes are under selection in *B*. *sinensis* indicates that the species still harbors diverse adaptive potential to potentially support its persistence.

Among 388 adaptive SNPs, BayPass identified 41 SNPs that were significantly associated with climate variables. RDA indicated that they were mainly correlated with short wave radiation (SWR) and long-wave radiation (LWR), in contrast to relative humidity (RH) and annual snowfall (AS). Both LWR and SWR are energy source and related to temperature.^140,141^ These results indicated that the significantly selected SNPs in climate were resistant to low humidity and high temperature. *B. sinensis* is a subtropical species that grows in a cool and humid climate, where it experiences frost but not excessive heat.^29^ The association of SNPs and populations in RDA (Figure 7B), which showed most populations clustered together along the RH and AS axis clearly confirmed this.

Nine of the 41 climate associated SNPs were identified in two genes (Figure 7A), BLS_040253-T1 and BLS_045871-T1. Annotation analysis indicated that these two genes were *SKIP*2 and *NAP*1 (Table S12). *SKIP*2 is an F-box family protein that is involved in the protein ubiquitination pathway. In plants, F-box proteins are one of the largest families, playing a pivotal role in plant growth and development and adverse conditions adaptation.^142,143^ *SKIP*2, in particular, is related to root development.^143^ Nuclesome assembly protein 1 (*NAP*1), belongs to a family of histone chaperones^144^. Because plants are sessile and vulnerable to stressful environments causing DNA damage, *NAP*1 combined with the other proteins modulates the repair of DNA damage, which is important to the maintenance of chromatin architecture facilitating plant genome stability and normal growth.^144,145^ However, how these SNPs alter the gene function promoting climate adaptation in *B. sinensis* needs further experimental confirmation.

We detected significant IBD for both adaptive and non-adaptive SNPs in overall populations and the western group, but not in the eastern group (Figure 7C, D). One possible reason for the lack of IBD in the eastern group could be attributed to spatial expansion and genetic interexchange among different refugia eroding the IBD effect. Furthermore, although adaptive SNPs are expected to correlate with environments or the other selection factors, they have also been observed following IBD in plants due to the correlation of geographic distance with selection factors.^146^ In the present study, for all climate variables except AS, we observed that they were significantly correlated with geographic distance (Mantel test, *P* < 0.05), suggesting that both isolation-by-distance and isolation-by-environment influenced the genetic diversities of *B*. *sinensis*.

1. **Conclusions**

As the sole species in the *Bretschneidera* genus and a Paleogene relic tree species, chromosome-level genome assembly and resequencing for adaptive SNP identification analysis provided important reference information for phylogeny, genetics, evolution, and the endangerment mechanism of *B. sinensis*. Demographic history dynamics revealed that the effective population size was influenced by historical events, and population genetic results will help in the study of current survival and evolutionary potential, species distribution, and migration.

### Data availability

We deposited the sequenced reads to NCBI Sequence Read Archive under the accession number SRR12656547 for the PacBio HiFi reads, SRR12666035 for the Illumina WGS reads, SRR12548980 for the Illumina Hi-C reads, SRR13013654 for the Illumina RNA-seq reads, SRR13013685 for the PacBio Iso-seq reads, and SRR14234257-SRR14234410 for the 154 resequencing reads. The high-quality, assembled genome was submitted to GenBank under the accession number GCA_018105755.1. The assembly, repeats and gene annotation, SNPs, and raw outputs for syntenic block, DupGen_finder and OrthoFinder are available at https://doi.org/10.6084/m9.figshare.15057996.v1.

**Acknowledgements**

We thank Xing-Liang Hou for helpful discussion. This work was supported by the National Natural Science Foundation of China ( No. 31570337); Key Special Project for Introduced Talents Team of Southern Marine Science and Engineering Guangdong Laboratory (Guangzhou) under Grant (No. GML2019ZD0408); Forestry Flora and Fauna Conservation Project of Guangdong Province: General regulations of "one center and three bases" for rare and endangered plant preservation in Guangdong Province and inventory of endemic plants and important wild south-China herbal medicinal resources in three regions; The project of Monitoring of Rare and Endemic Plants in Guangdong Province from Forest Resources Conservation Center of Guangdong Province; The Strategic Priority Research Program of the Chinese Academy of Sciences (XDA13020601).

### Authors’ contributions

H.F. C. and Z.F. W. designed the study. H.F. C. funded genome sequencing. Z.F. W. performed computational analyses. H.L. L., AJ H., Z.F. W., H.F. C., Z.A. L. and W. X. wrote the manuscript.

**Competing interests**

The authors declare that they have no competing interests.

**References**

1. Habel, J.C. and Assmann, T. 2010, *Relict Species: Phylogeography and Conservation Biology.* Springer-Verlag: Berlin Heidelberg.
2. Poncet, V., Munoz, F., Munzinger, J., Pillon, Y., et al. 2013, Phylogeography and niche modelling of the relict plant *Amborella trichopoda* (Amborellaceae) reveal multiple Pleistocene refugia in New Caledonia, *Mol. Ecol.*, **22**, 6163-6178.
3. Tang, C.Q., Matsui, T., Ohashi, H., et al. 2018, Identifying long-term stable refugia for relict plant species in East Asia, *Nat. Commun.*, **9**, 4488.
4. Zhao, Y.P., Fan, G.Y., Yin, P.P., et al. 2019, Resequencing 545 ginkgo genomes across the world reveals the evolutionary history of the living fossil, *Nat. Commun.*, **10**, 4201.
5. Toledo, B., Marcer, A., Mendez-Vigo, B., Alonso-Blanco, C. and Pico, F.X. 2020, An ecological history of the relict genetic lineage of *Arabidopsis thaliana*, *Environ. Exp. Bot.*, **170**, 103800.
6. Zhu, S.S., Chen, J., Zhao, J., et al. 2020, Genomic insights on the contribution of balancing selection and local adaptation to the long-term survival of a widespread living fossil tree, *Cercidiphyllum japonicum*, *New Phytol*., **228**, 1674-1689.
7. Li, M.J., Yang, Y.Z., Xu, R.P., et al. 2021, A chromosome-level genome assembly for the tertiary relict plant *Tetracentron sinense* oliv. (trochodendraceae), *Mol. Ecol. Resour.*, **21**, 1186-1199.
8. Yang, D.Q. and Hu, C.M. 1985, The chromosomes of *Bretschneidera* Hemsl, *Notes from the Royal Botanic Garden, Edinburgh*, **42**, 347-349.
9. Wolfe, J.A. 1975, Some aspects of plant geography in the northern hemisphere during the late Cretaceous and Tertiary, *Ann. Mo. Bot. Gard.*, **62**, 264-279.
10. Wang, M.N., Duan, L., Qiao, Q., et al. 2018, Phylogeography and conservation genetics of the rare and relict *Bretschneidera sinensis* (Akaniaceae), *PLoS One*, **13**, e0189034.
11. Xu, G.B., Liang, Y., Jiang, Y., et al. 2013, Genetic diversity and population structure of *Bretschneidera sinensis*, an endangered species, *Biodiversity Science*, **21**, 723-731.
12. Yu, Y.F. 1999, A milestone of wild plants protection in China-*The Catalogue of National Key Protected Wild Plants (Part I)*, *Plants*, **5**, 3-11.
13. Sun, W. 1998,*Bretschneidera sinensis*. *The IUCN Red List of Threatened Species network* <https://dx.doi.org/10.2305/IUCN.UK.1998.RLTS.T32324A9697750.en> (2021).
14. Qiao, Q., Xing, F.W., Chen, H.F. and Wang, M.N. 2011, Research progress and direction in *Bretschneidara sinensis*, a rare endemic plant in China, *Chinese Wild Plant Resources*, **30**, 4-8, 13.
15. Montaut, S., Zhang, W.D., Nuzillard, J.M., De Nicola, G.R. and Rollin, P. 2015, Glucosinolate diversity in *Bretschneidera sinensis* of Chinese origin, *J. Nat. Prod.*, **78**, 2001-2006.
16. The Angiosperm Phylogeny Group, et al. 2016, An update of the Angiosperm Phylogeny Group classification for the orders and families of flowering plants: APG IV, *Bot. J. Linn. Soc.*, **181**, 18.
17. Huang, S., Zhou, Q., Shen, H. Chen, Z., Zhu, Q. and Wu, W. 2018. The complete chloroplast genome of *Bretschneidera sinensis* (Bretschneideraceae). *Conserv. Genet. Resour.*, 10, 751-753.
18. Ma, D.X. and Liu R.L. 2012, Comparative study on germination of *Bretschneidera sinensis* seeds treated with solution extracted from forest litter and other methods, *Forest Res.*, **25**, 632-637.
19. Guo, X.Q., Zhou, L.H., Xiong, Z.H., et al. 2012, Study on the growth characteristic of young trees of *Bretschneidera sinensis*, *Subtropical Plant Sci.*, **41**, 32-36.
20. Zhang, J., Tian, H.L., Zhu, Y., et al. 2019, Research on endangered mechanism of *Bretschneidera sinensis*, *Modern Agr. Sci. Tech.*, **4**, 122-124, 127.
21. Liu, J.L., Xia, L.Z., Zheng, W.C., et al. 2015, Response of photosynthetic physiology of *Bretschneidera sinensis* seedlings to different soil water contents, *J. Cent. South Univ. Forest. Tech.*, **35**, 15-19.
22. Zhang, S., Chen, H.F., Liu, J.X., Liu, D.M., Yan, H.L. and Wang, F.G. 2019, Effects of drought on physiological responses of *Bretschneidera sinensis*, *Int. J. Agric. Biol.*, **22**, 545-552.
23. Qiao, Q., Wen, X.Y., Cheng, H.F. and Xing, F.W. 2010, Ecological anatomy of the root in *Bretschneidera sinensis*, a threatened endemic plant in China, *J. Wuhan Bot. Res.*, **28(5)**, 544-549.
24. Qiao, Q., Xing, F.W., Chen, H.F., and Fu, L. 2010. Leaf structural characteristics in *Bretschneidera sinensis*, a rare endemic plant in China. *J. Wuhan Bot. Res.*, **28(2)**, 229-233.
25. Tu, Q., Wu T., Zhao, L.C., Liu, Y.C., Liu, R.L. and Zhang, Z.X. 2012, Features of leaf appendages of *Bretschneidera sinensis* in different development stages, *Plant Diversity*, **34**, 248-256.
26. Qiao, Q., Chen, H.F., Xing, F.W., Wang, F.G. and Hou, X.G. 2012, Pollination ecology of *Bretschneidera sinensis* (Hemsley), a rare and endangered tree in China, *Pak. J. Bot.*, **44**, 1897-1903.
27. Qiao, Q., Li, T., Guo, X.F., Chen, H.F., Xing, F.W. 2020, Characterization of pollen and stigma of *Bretschneidera sinensis* Hemsl., a tertiary relict tree, *J Plant Genet. Resour.*, **21**, 1044-1050.
28. Qiao, Q., Xing, F.W., Chen, H.F., Fu, L. 2010, Leaf structural characteristics in *Bretschneidera sinensis*, a rare endemic plant in China, *J. Wuhan Bot. Res.*, **28**, 229-233.
29. Qiao, Q., Qin, X.S., Xing, F.W., Chen, H.F., Liu, D.M. 2011, Death causes and conservation strategies of the annual regenerated seedlings of rare plant, *Bretschneidera sinensis*, *Acta Ecologica Sinica*, **31**, 4709-4716.
30. Qiao, Q., Chen, H.F., Xing, F.W. 2009, Seed category and storage of *Bretschneidera sinensis*, a rare endemic plant in China, *Seed*, **28**, 25-27.
31. Yu, X.Y., Tian, H.L. and Guo, Z.Y. 2016, Community characteristics and interspecific relationship of *Bretschneidera sinensis* in southern Guizhou, China, *J. Sichuan Agr. Univ.*, **34**, 29-33.
32. Doyle, J.J. and Doyle, J.L. 1987, A rapid DNA isolation procedure for small quantities of fresh leaf tissue, *Phytochem. Bull.*, **19**, 11-15.
33. Długosz, M. and Deorowicz, S. 2017, Reckoner: read error corrector based on KMC, *Bioinformatics*, **33**, 1086-1089.
34. Chikhi, R. and Medvedev, P. 2014, Informed and automated *k*-mer size selection for genome assembly, *Bioinformatics*, **30**, 31-37.
35. Vurture, G.W., Sedlazeck, F.J., Nattestad, M., et al. 2017, GenomeScope: fast reference-free genome profiling from short reads, *Bioinformatics*, **33**, 2202-2204.
36. Guan, D.F., McCarthy, S.A., Wood, J., Howe, K. and Wang, Y.D. 2020, Identifying and removing haplotypic duplication in primary genome assemblies, *Bioinformatics*, **36**, 2896-2898.
37. Durand, N.C., Shamim, M.S., Machol, I., et al. 2016, Juicer provides a one-click system for analyzing loop-resolution Hi-C experiments, *Cell Syst.*, **3**, 95-98.
38. Dudchenko, O., Batra, S.S., Omer, A.D., et al. 2017, De novo assembly of the *Aedes aegypti* genome using Hi-C yields chromosome-length scaffolds, *Science*, **356**, 92-95.
39. James, T.R., Douglass, T., Neva, C.D., Helga, T., Jill, P.M. and Erez, L.A. 2018, Juicebox.js provides a cloud-based visualization system for Hi-C data*, Cell Syst.*, **6**, 256-258.
40. Xu, G.C., Xu, T.J., Zhu, R., et al. 2019, LR_Gapcloser: a tiling path-based gap closer that uses long reads to complete genome assembly, *Gigascience*, **8**,157.
41. Seppey, M., Manni, M. and Zdobnov, E.M. 2019, BUSCO: Assessing Genome Assembly and Annotation Completeness, *In Gene Prediction: Methods and Protocols*, edited by M. Kollmar, Methods in Molecular Biology, 227-245.
42. Yang, L.A., Chang, Y.J., Chen, S.H., Lin, C.Y. and Ho, J.M. 2019, SQUAT: a sequencing quality assessment tool for data quality assessments of genome assemblies, *BMC Genomics*, **19**, 238.
43. Ou, S.J., Su, W.J, Liao, Y., et al. 2019, Benchmarking transposable element annotation methods for creation of a streamlined, comprehensive pipeline, *Genome Biol.*, **20**, 275.
44. Girgis, H.Z. 2015, Red: an intelligent, rapid, accurate tool for detecting repeats de-novo on the genomic scale, *BMC Bioinformatics*, **16**, 227.
45. Quinlan, A.R. and Hall, I.M. 2010, BEDTools: a flexible suite of utilities for comparing genomic features, *Bioinformatics*, **26**, 841-842.
46. Cook, D.E., Valle-Inclan, J.E., Pajoro, A., Rovenich, H., Thomma, B.P.H.J. and Faino, L. 2019, Long-Read Annotation: Automated eukaryotic genome annotation based on long-read cDNA sequencing, *Plant Physiol.*, **179**, 38-54.
47. Zhang, H., Tanner, Y., Huang, L. and Entwistle, S. 2018, dbCAN2: a meta server for automated carbohydrate-active enzyme annotation, *Nucleic Acids Res.*, **46**, W95-W101.
48. Huerta-Cepas, J., Forslund, K., Coelho, L.P., et al. 2017, Fast genome-wide functional annotation through orthology assignment by eggNOG-mapper, *Mol. Biol. Evol.*, **34**, 2115-2122.
49. Ashburner, M., Ball, C.A., Blake, J.A., et al. 2000, Gene ontology: tool for the unification of biology, *Nat. Genet.*, **25**, 25-29.
50. The Gene Ontology Consortium. 2019, The Gene Ontology Resource: 20 years and still GOing strong, *Nucleic Acids Res.*, **47**, D330-D338.
51. Kanehisa, M., Soto, Y., Kawashima, M., Furumichi, M. and Tanabe, M. 2016, KEGG as a reference resource for gene and protein annotation, *Nucleic Acids Res.*, **44**, 457-462.
52. Mitchell, A.L., Attwood, T.K., Babbitt, P.C., et al. 2019, InterPro in 2019: improving coverage, classification and access to protein sequence annotationsn, *Nucleic Acids Res.*, **47**, D351-D360.
53. Rawlings, N.D., Barrett, A.J., Thomas, P.D., Huang, X., Bateman, A. and Finn, R.D. 2018, The merops database of proteolytic enzymes, their substrates and inhibitors in 2017 and a comparison with peptidases in the PANTHER database, *Nucleic Acids Res.*, **46**, D624-D632.
54. El-Gebali, S., Mistry, J., Bateman, A., et al. 2019, The Pfam protein families database in 2019, *Nucleic Acids Res.*, **47**, D427-D432.
55. The UniProt Consortium. 2019, UniProt: a worldwide hub of protein knowledge, *Nucleic Acids Res.*, **47**, D506-D515.
56. Petersen, T.N., Brunak, S., von-Heijne, G. and Nielsen, H. 2011, SignalP 4.0: discriminating signal peptides from transmembrane regions, *Nat. Methods*, **8**, 785-786.
57. Emms, D.M. and Kelly, S. 2015, OrthoFinder: solving fundamental biases in whole genome comparisons dramatically improves orthogroup inference accuracy, *Genome Biol.*, **16**, 157.
58. Emms, D.M. and Kelly, S. 2019, OrthoFinder: phylogenetic orthology inference for comparative genomics, *Genome Biol.*, **20**, 238.
59. Sanderson, M.J. 2002, Estimating absolute rates of molecular evolution and divergence times: a penalized likelihood approach, *Mol. Biol. Evol.*, **19**, 101-109.
60. Smith, S.A. and O'Meara, B.C. 2012, Divergence time estimation using penalized likelihood for large phylogenies, *Bioinformatics*, 28, 2689-2690.
61. Han, M.V., Thomas, G.W.C., Jose, L.M. and Hahn M.W. 2013, Estimating gene gain and loss rates in the presence of error in genome assembly and annotation using cafe 3, *Mol. Biol. Evol.*, **30**, 1987-1997.
62. Chen, C.J., Chen, H., Zhang, Y., Thomas, H.R., Frank, M.H., He, Y.H. and Xia, R. 2020, TBtools: an integrative toolkit developed for interactive analyses of big biological data, *Mol. Plant*, **13**, 1194-1202.
63. Tian, T., Liu, Y., Yan, H.Y., et al. 2017, agriGO v2.0: a GO analysis toolkit for the agricultural community, 2017 update, *Nucleic Acids Res.*, **45**, W122-W129.
64. Supek, F., Bosnjak, M., Skunca, N., Smuc, T. 2011, REVIGO summarizesand visualizes long lists of gene ontology terms, *PLoS One*, **6**, e21800.
65. Benjamini, Y. and Hochberg, Y. 1995, Controlling the false discovery rate: a practical and powerful approach to multiple testing, *J. Roy. Stat. Soc. (Series B)*, **57**, 289-300.
66. Qiao, X., Li, Q.H., Yin, H., et al. 2019, Gene duplication and evolution in recurring polyploidization-diploidization cycles in plants, *Genome Biol.*, **20**, 38.
67. Tang, H.B., Bowers, J.E., Wang, X.Y., Ming, R., Alam, M. and Paterson, A.H. 2008, Synteny and collinearity in plant genomes, *Science*, **320**, 486-488.
68. Tang, H.B., Krishnakumar, V., Li, J.P., Zhang, X.T. 2015, jcvi: JCVI utility libraries, https://zenodo.org/record/31631#.YQljC8gaWUk.
69. Yu, Y.M., Ou yang, Y.D. and Yao, W. 2018, shinyCircos: an R/Shiny application for interactive creation of Circos plot, *Bioinformatics*, **34**, 1229-1231.
70. Schiffels, S. and Durbin, R. 2014, Inferring human population size and separation history from multiple genome sequences, *Nat. Genet.*, **46**, 919-925.
71. Millar, C.D., Dodd, A., Anderson, J., Gibb, G.C., Ritchie, P.A., Baroni, C., et al. 2008, Mutation and evolutionary rates in *Adélie Penguins* from the Antarctic, *PLoS Genetics*, **4**, e1000209.
72. Jukes, T.H. and Cantor, C.R. 1969, *Evolution of protein molecules*. *Mammalian protein metabolism,* Academic Press: New York.
73. Patil, A.B. and Vijay, N. 2021, Repetitive genomic regions and the inference of demographic history, *Heredity*, **127**, 151-166.
74. Puritz, J.B., Hollenbeck, C.M. and Gold, J.R. 2014, dDocent: a RADseq, variant-calling pipeline designed for population genomics of non-model organisms, *PeerJ*, **2**, e431.
75. Purcell, S., Neale, B., Todd-Brown, K., et al. 2007, PLINK: a tool set for whole-ge-nome association and population-based linkage analyses, *Ame. J. Hum. Genet.*, **81**, 559-575.
76. Alexander, D.H., Novembre, J. and Lange, K. 2009, Fast model-based estimation of ancestry in unrelated individuals, *Genome Res.*, **19**, 1655-1664.
77. Zheng, X.W., Levine D., Shen, J., Gogarten, S.M., Laurie, C. and Weir, B.S. 2012, A high-performance computing toolset for relatedness and principal component analysis of SNP data, *Bioinformatics*, **28**, 3326-3328.
78. Mussmann, S., Douglas, M.R., Chafin, T. and Douglas, M.E. 2020, AdmixPipe: population analyses in Admixture for non-model organisms, *BMC Bioinformatics*, **21**, 337.
79. Evanno, G., Regnaut, S. and Goudet, J. 2005, Detecting the number of clusters of individuals using the software STRUCTURE: a simulation study, *Mol. Ecol.*, **14**, 2611-2620.
80. Kopelman, N.M., Mayzel, J., Jakobsson, M., Rosenberg, N.A. and Mayrose, I. 2015, Clumpak: a program for identifying clustering modes and packaging population structure inferences across *K*, *Mol. Ecol. Resour.*, **15**, 1179-1191.
81. Luu, K., Bazin, E. and Blum, M.G. 2017, pcadapt: an R package to perform genome scans for selection based on principal component analysis, *Mol. Ecol. Resour.*, **17**, 67-77.
82. Privé, F., Luu, K., Vilhjálmsson, B.J. and Blum, M.G.B. 2020, Performing highly efficient genome scans for local adaptation with R package pcadapt version 4, *Mol. Biol. Evol.*, **37**, 2153-2154.
83. Gautier, M. 2015, Genome-wide scan for adaptive divergence and association with population-specific covariates, *Genetics*, **201**, 1555-1579.
84. Günther, T., Coop, G. 2013, Robust identification of local adaptation from allele frequencies, *Genetics*, **195**, 205-220.
85. Amy McNally NASA/GSFC/HSL. 2018, FLDAS Noah Land Surface Model L4 Global Monthly 0.1 x 0.1 degree (MERRA-2 and CHIRPS), Greenbelt, MD, USA, *Goddard Earth Sciences Data and Information Services Center* (GES DISC), Accessed: [2021-12-20], 10.5067/5NHC22T9375.
86. Oksanen, J., Blanchet, F.G., Friendly, M., et al. 2020, vegan: Community Ecology Package. R package version 2.5-7, https://CRAN.R-project.org/package=vegan
87. Bohonak, A.J. 2002, IBD (Isolation By Distance): a program for analyses of isolation by distance, *J. Hered.*, **93**, 153-154.
88. Korunes, K.L. and Samuk, K. 2021, pixy: Unbiased estimation of nucleotide diversity and divergence in the presence of missing data, *Mol. Ecol. Resour.*, **21**, 1359-1368.
89. Ming, R., Hou, S.B., Feng, Y., et al. 2008, The draft genome of the transgenic tropical fruit tree papaya (*Carica papaya* Linnaeus), *Nature*, **452**, 991-996.
90. Quesneville, H. 2020, Twenty years of transposable element analysis in the *Arabidopsis thaliana* genome, *Mobile DNA*, **11**, 28.
91. Su, W., Gu, X. and Peterson, T. 2019, TIR-Learner, a new ensemble method for TIR transposable element annotation, provides evidence for abundant new transposable elements in the maize genome. *Molecular Plant*, **12**, 447-460.
92. Afzal, A.J., Wood, A.J. and Lightfoot, D.A. 2008, Plant receptor-like serine threonine kinases: roles in signaling and plant defense, *Mol. Plant-Microbe In.*, **21**, 507-517.
93. Wang, Y.P., Zhou, Z.J., Gao, J.Y., et al. 2016, The mechanisms of maize resistance to *Fusarium verticillioides* by comprehensive analysis of RNA-seq data, *Front. Plant Sci.*, **7**, 1654.
94. Yang, X.H., Guo, X., Yang, Y., et al. 2018, Gene profiling in late blight resistance in potato genotype SD20, *Int. J. Mol. Sci.*, **19**, 1728.
95. Feng, H., Li, H.L., Zhang, M., et al. 2019, Responses of Fuji (*Malus domestica*) and Shandingzi (*Malus baccata*) apples to Marssonina coronaria infection revealed by comparative transcriptome analysis, *Physiol. Mol. Plant P.*, **106**, 87-95.
96. Lv, T.X., Li, X.M., Fan, T., et al. 2019, The calmodu-lin-binding protein IQM1 interacts with CATALASE2 to affect pathogen defense, *Plant Physiol.*, **181**, 1314-1327.
97. Sucher, J., Mbengue, M., Dresen, A., et al. 2020, Phylotranscriptomics of the pentapetalae reveals frequent regulatory variation in plant local responses to the fungal pathogen sclerotinia sclerotiorum, *The Plant Cell*, **32**, 1820-1844.
98. Pogoda, C.S., Reinert, S., Talukder, Z.I., et al. 2021, Genetic loci underlying quantitative resistance to necrotrophic pathogens *Sclerotinia* and *Diaporthe* (*Phomopsis*), and correlated resistance to both pathogens, *Theor. Appl. Genet.*, **134**, 249-259.
99. Smakowska-Luzan, E., Mott, G.A., Parys, K., et al. 2018, An extracellular network of *Arabidopsis* leucine-rich repeat receptor kinases, *Nature*, **553**, 342-346.
100. Govrin, E.M. and Levine, A. 2000, The hypersensitive response facilitates plant infection by the necrotrophic pathogen *Botrytis cinerea*, *Current Biology*, **10**, 751-757.
101. Balint-Kurti, P. 2019, The plant hypersensitive response: concepts, control and consequences, *Mol. Plant Pathol.*, **20**, 1163-1178.
102. Zhang, S.Q. and Klessig, D.F. 2001, MAPK cascades in plant defense signaling, *Trends Plant Sci.*, **6**, 520-527.
103. Meng, X.Z. and Zhang, S.Q. 2013, MAPK cascades in plant disease resistance signaling, *Annu. rev. Phytopathol.*, **51**, 245-266.
104. Bigeard, J. and Hirt, H. 2018, Nuclear Signaling of Plant MAPKs, *Front. Plant Sci.*, **9**, 469.
105. Huang, X.X., Zhu, G.Q., Liu, Q., Chen, L., Li, Y.J. and Hou, B.K. 2018, Modulation of plant salicylic acid-associated immune responses via glycosylation of dihydroxybenzoic acids, *Plant Physiol.*, **176**, 3103-3119.
106. Jiang, S.Y., Jin, J.J., Sarojam, R. and Ramachandran, S. 2019, A comprehensive survey on the terpene synthase gene family provides new insight into its evolutionary patterns, *Genome Biol. Evol.*, **11**, 2078-2098.
107. Chen, F., Tholl, D., Bohlmann, J. and Pichersky, E. 2011, The family of terpene synthases in plants: a mid-size family of genes for specialized metabolism that is highly diversified throughout the kingdom, *Plant J.*, **66**, 212-229.
108. Pichersky, E. and Raguso, R.A. 2016, Why do plants produce so many terpenoid compounds? *New Phytol.*, **220**, 655-658.
109. Thrash, A., Hoffmann, F., Perkins, A. 2020, Toward a more holistic method of genome assembly assessment, *BMC Bioinformatics*, **21**(Suppl 4), 249.
110. Lin, Y., Yuan, J., Kolmogorov, M., Shen, M. W., Chaisson, M. and Pevzner, P. 2016, Assembly of long error-prone reads using de Bruijn Graphs, *P. Natl Acad Sci USA*, **113**, E8396-E8405
111. Kolmogorov, M., Yuan, J., Lin, Y. and Pevzner, P. 2019, Assembly of long error-prone reads using repeat graphs, *Nature Biotech.*, **37**, 540-546.
112. Prothero, D.R. 1994, *The Eocene-Oligocene Transition: Paradise Lost*, Columbia University Press: New York.
113. Zachos, J., Pagani, M., Sloan, L., Thomas, E. and Billups, K. 2001, Trends, rhythms, and aberrations in global climate 65 Ma to present, *Science*, **292**, 686-693.
114. Liu, Z.H., Pagani, M., Zinniker, D., et al. 2009, Global cooling during the eocene-oligocene climate transition, *Science*, **323**, 1187-1190.
115. Zhang, Q.Q., Smith, T., Yang, J. and Li, C.S. 2016, Evidence of a cooler continental climate in East China during the warm Early Cenozoic, *PLoS One*, **11**, e0155507.
116. Tang, H., Li, S.F., Su, T., et al. 2020, Early Oligocene vegetation and climate of southwestern China inferred from palynology, *Palaeogeog. Palaecol.*, **560**, 109988.
117. Zheng, B.X., Xu, Q.Q. and Shen, Y.P. 2002, The relationship between climate change and Quaternary glacial cycles on the Qinghai-Tibetan Plateau: review and speculation, *Quatern. Int.*, **97-98**, 93-101.
118. Beichman, A.C., Phung, T.N. and Lohmueller, K.E. 2017, Comparison of single genome and allele frequency data reveals discordant demographic histories, *G3: Genes Genom. Genet.*, **7**, 3605-3620.
119. Yao, Q.Y. 1980, An approach of the formation of Qiongzhou Strait, *Trop. Geogr. Q.*, **1**, 21-25.
120. Zhao, Z.B. 1982, A preliminary study on the evolution of Taiwan Strait, *Taiwan Strait*, **1**, 20-24.
121. Li, G.X. 1994, The formation and evolution of Cenozoic rifts in Taiwan Strait, *Trop. Oceanol.*, **13**, 18-24.
122. Yang, X.Q., Song, W.L. and Chen, C.H. 1996, Characteristics of geological structure in Taiwan Strait, *Journal of Oceanography in Taiwan Strait*, **15**, 127-136.
123. Chen, L., Zhang, Y., Li, T., Yang, W. and Chen, J. 2014, Sedimentary environment and its evolution of Qiongzhou Strait and nearby seas since last ten thousand years, *Earth Sci.*, **39**, 696-704.
124. Ni, Y.G., Xia, Z. and Ma, S.Z. 2014, The opening of Qiongzhou Strait: evicence from sub-bottom profiles, *Mar. Geol. Quatern. Geol.*, **34**, 79-82.
125. Huang, Q.Y. 2017, Geological ages of Taiwan stratigraphy and tectonic events, *Scientia Sinica Terrae*, **47**, 394-405.
126. Papuga, J., Hoffmann, C., Dieterle, M., et al. 2010, Arabidopsis LIM proteins: a family of actin bundlers with distinct expression patterns and modes of regulation, *The Plant Cell,* **22**, 3034-3052.
127. Ye, J.R., Zhou, L.M. and Xu, M.L. 2013, *Arabidopsis* LIM proteins PLIM2a and PLIM2b regulate actin configuration during pollen tube growth, *Biol. Plantarum*, **57**, 433-441.
128. Jia, N., Liu, X.M. and Gao, H.B. 2016, A DNA2 homolog is required for DNA damage repair, cell cycle regulation, and meristem maintenance in plants, *Plant Physiol.*, **171**, 318-333.
129. Yang, Y., Xu, R., Ma, C.J., Vlot, A.C., Klessig, D.F. and Pichersky, E. 2008, Inactive methyl indole-3-acetic acid ester can be hydrolyzedand activated by several esterases belonging to the *At*MES ester-ase family of *Arabidopsis*, *Plant Physiol.*, **147**, 1034-1045.
130. Woodward, A.W. and Bartel, B. 2005, Auxin: regulation, action, and interaction, *Ann. Bot-London*, **95**, 707-735.
131. Enders, T.A. and Strader, L.C. 2015, Auxin activity: past, present, and future, *Am. J. Bot.*, **102**, 180-196.
132. Fujimoto, T., Abe, H., Mizukubo, T. and Seo, S. 2021, Phytol, a constituent of chlorophyll, induces root-knot nematode resistance in *Arabidopsis* via the ethylene signaling pathway, *Mol. Plant-Microbe In.*, **34**, 279-285.
133. Spicher, L., Almeida, J., Gutbrod, K., et al. 2017, Essen-tial role for phytol kinase and tocopherol in tolerance to combined light and temperature stress in tomato, *J. Exp. Bot.*, **68**, 5845-5856.
134. Aubourg, S., Kreis, M. and Lecharny, A. 1999, The DEAD box RNA helicase family in *Arabidopsis thaliana*, *Nucleic Acids Res.*, **27**, 628-636.
135. Zhang, X.M., Zhao, X.Q., Feng, C.X., et al. 2014, The cloning and characterization of a DEAD-box RNA helicase from stress-responsive wheat, *Physiol. Mol. Plant P.*, **88**, 36-42.
136. Baruah, I., Debbarma, J., Boruah, H.P.D. and Keshavaiah, C. 2017, The DEAD-box RNA helicases and multiple abiotic stresses in plants: a systematic review of recent advances and challenges, *Plant Omics*, **10**, 252-262.
137. Cavallari, N., Nibau, C., Fuchs, A., Dadarou, D., Barta, A. and,Doonan, J. H. 2018, The cyclin-dependent kinase G group defines a thermo-sensitive alternative splicing circuit modulating the expression of Arabidopsis ATU2AF65A, *Plant J.*, 94, 1010-1022.
138. Nibau, C., Dadarou, D., Kargios, N., et al. 2020, A functional kinase is necessary for cyclin-dependent kinase G1 (CDKG1) to maintain fertility at high ambient temperature in *Arabidopsis*, *Front. Plant Sci.*, **11**, 586870.
139. Nibau, C., Gallemí, M., Dadarou, D., Doonan, J.H. and Cavallari, N. 2020, Thermo-sensitive alterna-tive splicing of *FLOWERING LOCUS M* is modulated by cyclin-dependent kinase G2, *Front. Plant Sci.*, **10**, 1680.
140. Klassen, S., Bugbee, B. 2005, Shortwave Radiation, *Micrometeorol. Agr. Syst.*, **47** (Chapter 3), 43-57.
141. Jones, H. G., Rotenberg, E. 2011, Energy, radiation and temperature regulation in plants, <https://doi.org/10.1002/9780470015902.a0003199.pub2>
142. Zhang, T. C., Qiao, Q., Novikova P. Y., et al. 2019, Genome of *Crucihimalaya himalaica*, a close relative of *Arabidopsis*, shows ecological adaptation to high altitude, *P. Natl Acad Sci USA*, **116**, 7137-7146.
143. Abd‑Hamid, N. A., Ahmad‑Fauzi, M.I., Zainal, Z. and Ismail, I. 2020, Diverse and dynamic roles of F‑box proteins in plant biology, *Planta*, **251**, 68.
144. Tripathi, A. k., Singh, K., Pareek, A. and Singla-Pareek, S.L. 2015, Histone chaperones in *Arabidopsis* and rice: genome-wide identification, phylogeny, architecture and transcriptional regulation, *BMC Plant Biol.*, **15**, 42.
145. Banerjee, S. and Roy, S. 2021, An insight into understanding the coupling between homologous recombination mediated DNA repair and chromatin remodeling mechanisms in plant genome: an update, *Cell Cycle*, **20**, 1760-1784.
146. Shih, K.M., Chang, C.T., Chung, J.D., Chiang, Y.C. and Hwang S.Y. 2018, Adaptive genetic divergence despite significant isolation-by-distance in populations of Taiwan cow-tail fir (*Keteleeria davidiana var. formosana*), *Front. Plant Sci.*, **9**, 92.

**Table 1**. Sampling and climate information for 13 *Bretschneidera sinensis* populations in China. Climate variables are averaged from year 1982 to 2020 as downloaded from https://disc.gsfc.nasa.gov

| Population | Abbreviation | Longitude | Latitude | Sample size | AMT (℃) | RH (%) | AP (mm) | E (mm) | AS (mm) | LWR (W/m^2^) | SWR (W/m^2^) |
| --- | --- | --- | --- | --- | --- | --- | --- | --- | --- | --- | --- |
| Enshi | ES | 109˚08’01" | 30˚02'54" | 6 | 12.066 | 86.004 | 1413.520 | 1022.056 | 45.793 | 315.045 | 185.184 |
| Chongyi | CY | 114˚18’30" | 25˚40'55" | 15 | 16.835 | 85.914 | 1617.485 | 1202.128 | 28.841 | 346.931 | 195.432 |
| Jingning | JN | 119˚38’09" | 27˚58'24" | 15 | 15.289 | 88.085 | 2152.491 | 1226.724 | 38.463 | 340.070 | 193.905 |
| Longquan | LQ | 119˚08’29" | 28˚04'29" | 6 | 17.425 | 86.121 | 1821.218 | 1253.450 | 12.913 | 351.562 | 192.497 |
| Nanping | NP | 118˚10’39" | 26˚38'30" | 15 | 18.612 | 82.797 | 1809.063 | 1255.184 | 2.377 | 360.926 | 188.629 |
| Huizhou | HZ | 113˚52’06" | 23˚37'57" | 13 | 17.508 | 81.551 | 2249.696 | 1266.683 | 6.929 | 347.709 | 199.541 |
| Longsheng | LS | 109˚55’11" | 25˚37'31" | 15 | 14.477 | 85.583 | 1896.454 | 1169.204 | 50.396 | 332.110 | 198.686 |
| Jinxiu | JX | 110˚12’42" | 24˚09'35" | 15 | 14.594 | 82.132 | 1694.328 | 1138.587 | 33.598 | 329.956 | 201.576 |
| Liping | LP | 109˚08’12" | 26˚13'49" | 15 | 16.124 | 86.940 | 1425.229 | 1152.270 | 26.413 | 343.832 | 192.702 |
| Pingbian | PB | 103˚41’15" | 22˚59’01" | 8 | 16.874 | 78.295 | 1589.989 | 1195.990 | 1.225 | 338.621 | 223.474 |
| Zhangjiajie | ZJJ | 109˚41’45" | 29˚39'18" | 15 | 11.956 | 86.213 | 1472.285 | 1027.856 | 64.263 | 315.403 | 190.303 |
| Taibei | TB | 121˚51’34" | 25˚07'34" | 10 | 22.234 | 83.709 | 2404.350 | 1474.141 | 0.000 | 384.059 | 180.040 |
| Wuzhishan | WZS | 109˚23’19" | 18˚58'18" | 11 | 20.126 | 81.715 | 1874.228 | 1155.587 | 0.000 | 364.364 | 214.244 |
|  |  |  | Total | 154 |  |  |  |  |  |  |  |

AMT: annual mean temperature; RH: relative humidity; AP: annual precipitation; E: Evapotranspiration; AS: annual snowfall; LWR: long wave radiation; SWR: short wave radiation


**Table 2.** Statistics of the *Bretschneidera sinensis* genome assembly

| Statistics of HiFi assembly | |  | Statistics of the Hi-C scaffolded assembly | | | |  |
| --- | --- | --- | --- | --- | --- | --- | --- |
| Sequence length (bp) | Order of sequence length |  | Sequence length (bp) | Order of sequence length | | Sequence length in the genome |  |
| N10=64,553,695 | L10=2 |  | N10=152,689,475 | L10=1 | | 13.04% |  |
| N20=54,398,357 | L20=4 |  | N20=151,999,468 | L20=2 | | 12.98% |  |
| N30=48,448,289 | L30=6 |  | N30=144,368,824 | L30=3 | | 12.33% |  |
| N40=29,147,647 | L40=10 |  | N40=140,467,444 | L40=4 | | 12.00% |  |
| N50=24,630,967 | L50=14 |  | N50=137,464,959 | L50=5 | | 11.74% |  |
| N60=19,150,349 | L60=20 |  | N60=103,046,003 | L60=6 | | 8.80% |  |
| N70=14,033,915 | L70=28 |  | N70=102,104,410 | L70=7 | | 8.72% |  |
| N80=9,569,185 | L80=38 |  | N80=101,254,378 | L80=8 | | 8.65% |  |
| N90=3,151,252 | L90=58 |  | N90=87,593,448 | L90=9 | | 7.48% |  |
| N100=10,839 | L100=1,416 |  | N100=1,000 | L100=1,621 | |  |  |
| Total length | 1,222,797,721 bp |  | 1,170,884,685 bp |  |  | |  |
| Average | 863,557.71 bp |  | 722,322.45 bp |  |  | |  |
| Largest | 89,979,610 bp |  | 152,689,475 bp |  |  | |  |
| Shortest | 10,839 bp |  | 1,000 bp |  |  | |  |

**Figure legends**

**Figure 1** Representative photographs of *Bretschneidera sinensis*, including the whole tree, flowers, and fruits.

**Figure 2** Sampling locations and population structures in *Bretschneidera sinensis*. A) Geographic distribution of 13 *B*. *sinensis* populations in China; B) First two components of a principal component analysis of SNPs; C）ADMIXTURE results for *K* = 2.

**Figure 3** Features of the genome assembly A) Hi-C interaction heat map (bin length 210000 bp) for the *Bretschneidera sinensis* genome; B) Genome features across nine inferred chromosomes of *B*. *sinensis*. The gray-shaded tracks represent nine assembled chromosomes (scale: Mbp); green represents repeat density; red represents gene density, and dark blue represents SNP density; and the cyan lines inside the circle represent syntenic blocks. The densities of repeats, genes, and SNPs were estimated based on a 10^6^ bp window.

**Figure 4** A phylogenetic tree with mapped gene family contractions(–) and expansions(+) in *Bretschneidera sinensis* and other species. The number at the root denotes the total gene families within the most recent common ancestor (MRCA). Please note that not all gene family contractions or expansions presented in the tree are significant. A time scale (in millions of years) is shown beneath the tree. Numbers of identified terpene synthase genes with their Pfam ID PF01397 and PF03936 in malvids species are shown on the left of the tree.

**Figure 5** Gene ontology (GO) enrichment analysis of the genes in significantly expanded gene families.

**Figure 6** Demographic history of *Bretschneidera sinensis* showing the historical changes in effective population size. Arrows indicate large declines in population size that are described in the text.

**Figure 7.** Redundancy analysis (RDA) showing the association of climate variables with A) SNPs and B) individuals. The vectors indicate the climate variables of the first two RDA components. Isolation by distance (IBD) showing the association between genetic differentiation (*Fst*) and geographic distance using C) non-adaptive and D) adaptive SNPs in *Bretschneidera sinensis*. “**” means *P* < 0.01.

**Supplementary figure 1** Treemap showing hierarchy for GO terms enriched with expanded genes in biological process.

**Supplementary figure 2** Directed acyclic graph (DAG) of GO terms enriched with expanded genes in biological process. The yellow to red represent the gradient from low to high significance denoted by the adjusted P-value after GO term number in each colored box.

**Supplementary figure 3** Directed acyclic graph (DAG) of GO terms enriched with expanded genes in molecular function. The yellow to red represent the gradient from low to high significance denoted by the adjusted P-value after the GO term in each colored box.

**Supplementary figure 4** Directed acyclic graph (DAG) of GO terms over-represented for *Bretschneidera sinensis* in molecular function. The yellow to red represent the gradient from low to high significance denoted by the adjusted P-value after the GO term in each colored box.

**Supplementary figure 5** Directed acyclic graph (DAG) of GO terms under-represented for *Bretschneidera sinensis* in molecular function. The yellow to red represent the gradient from low to high significance denoted by the adjusted P-value after the GO term in each colored box.

**Supplementary figure 6**  Determining the optimal cluster number (*K*) inferred from ADMIXTURE; A) CV error and B) Δ*K* for each K
