# Supplementary figures and images for: The genome of the Paleogene relic tree Bretschneidera sinensis: insights into trade-offs in gene family evolution, demographic history, and adaptive SNPs
Source: DNA Res. 2022 Feb 4;29(1):dsac003. doi: 10.1093/dnares/dsac003 (PMC8825261; doi:10.1093/dnares/dsac003)

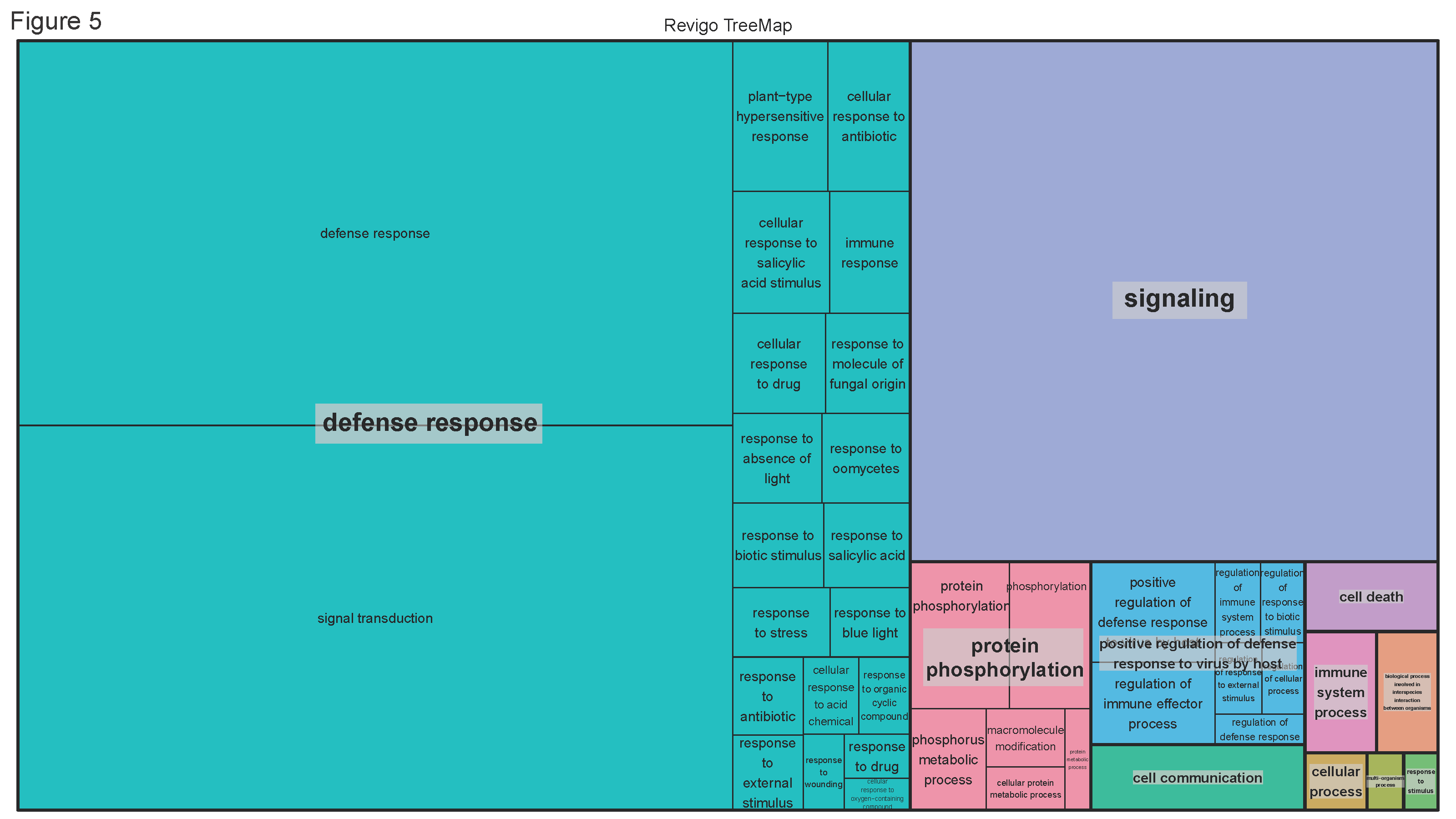

Supplement: dsac003_Supplementary_Data [file dsac003_supplementary_data.zip › Supplementary Figure 1.tiff]

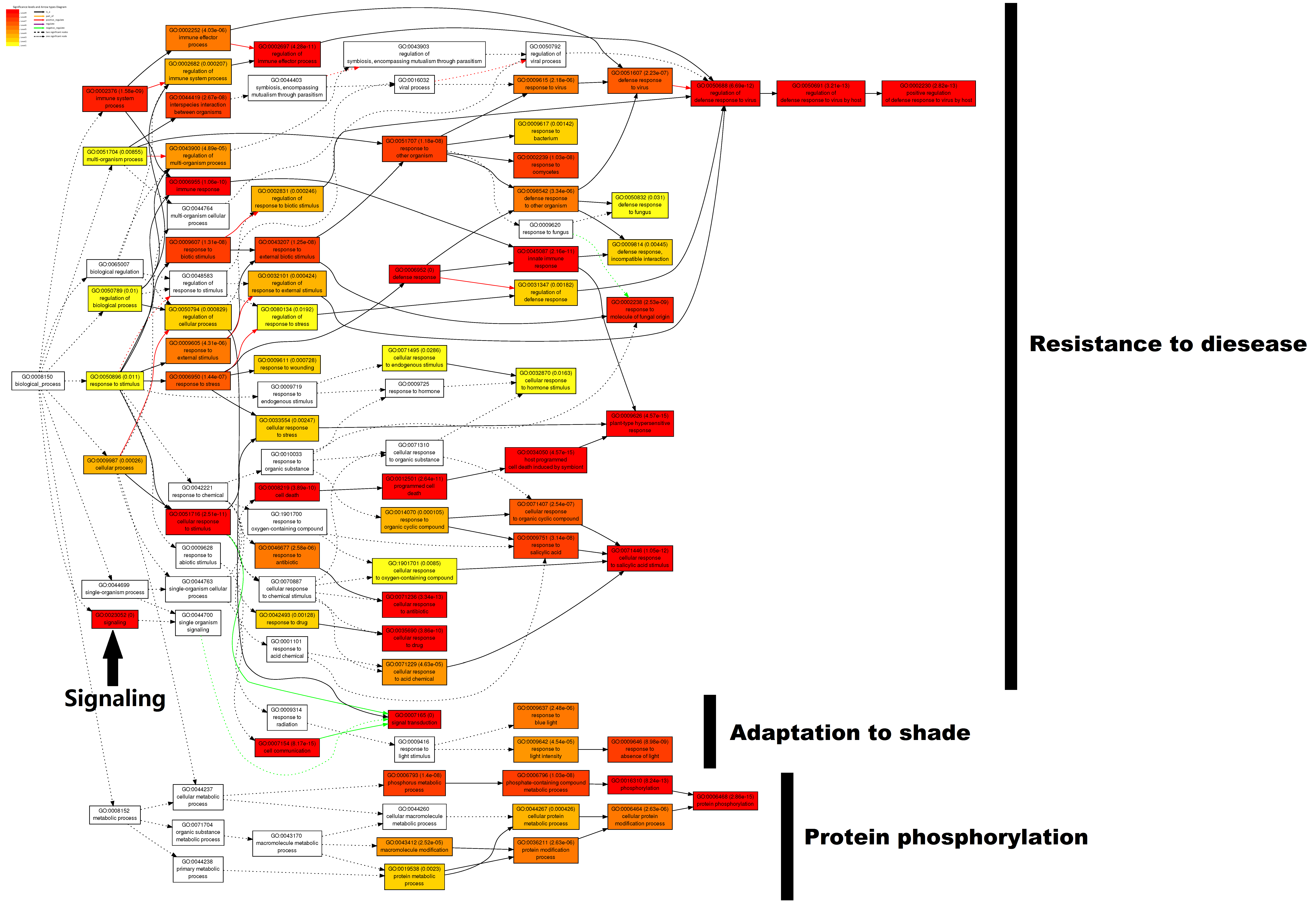

Supplement: dsac003_Supplementary_Data [file dsac003_supplementary_data.zip › Supplementary Figure 2.tif]

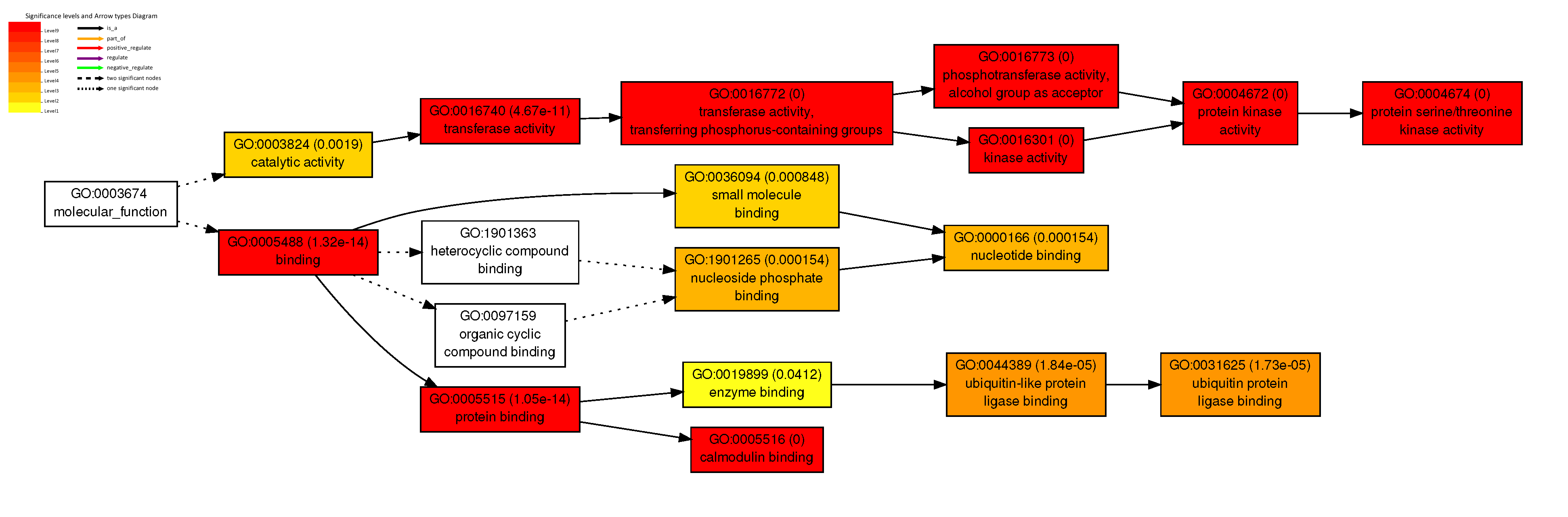

Supplement: dsac003_Supplementary_Data [file dsac003_supplementary_data.zip › Supplementary Figure 3.tif]

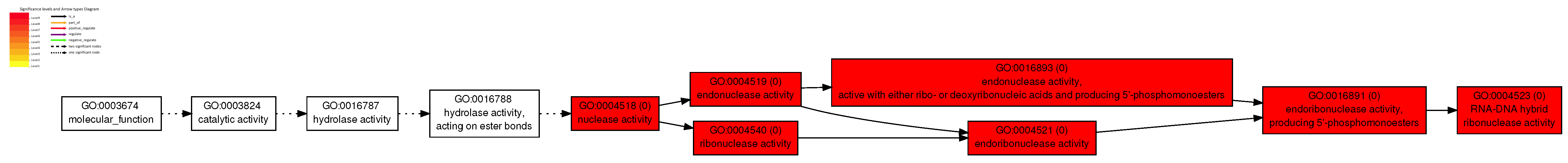

Supplement: dsac003_Supplementary_Data [file dsac003_supplementary_data.zip › Supplementary Figure 4.tif]

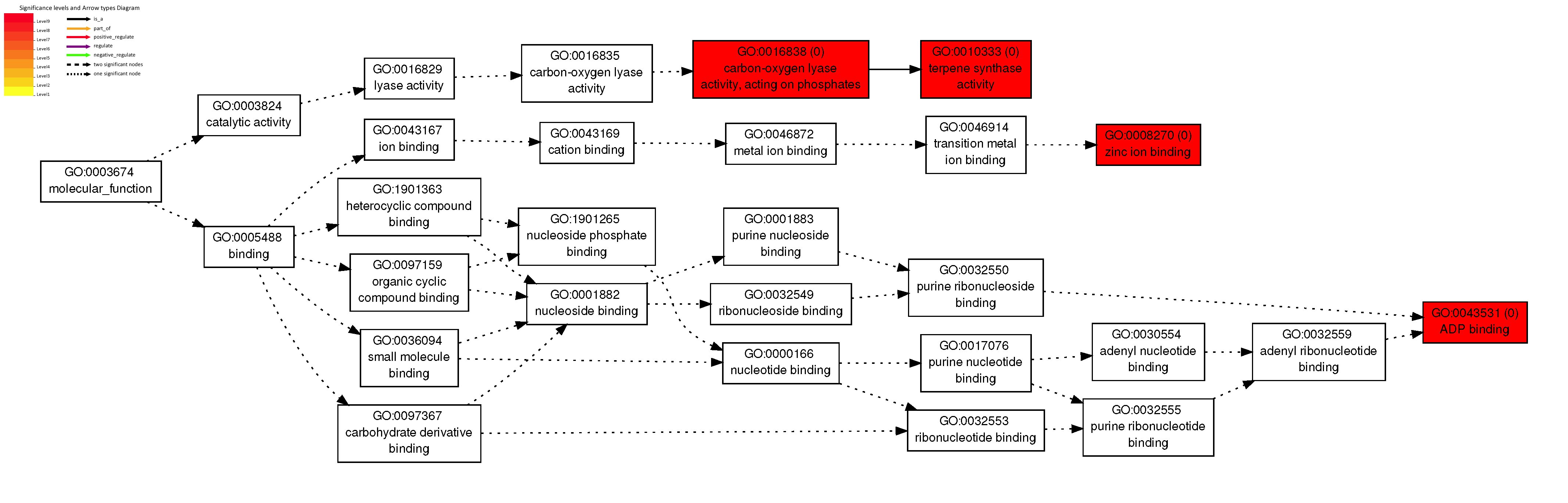

Supplement: dsac003_Supplementary_Data [file dsac003_supplementary_data.zip › Supplementary Figure 5.tif]

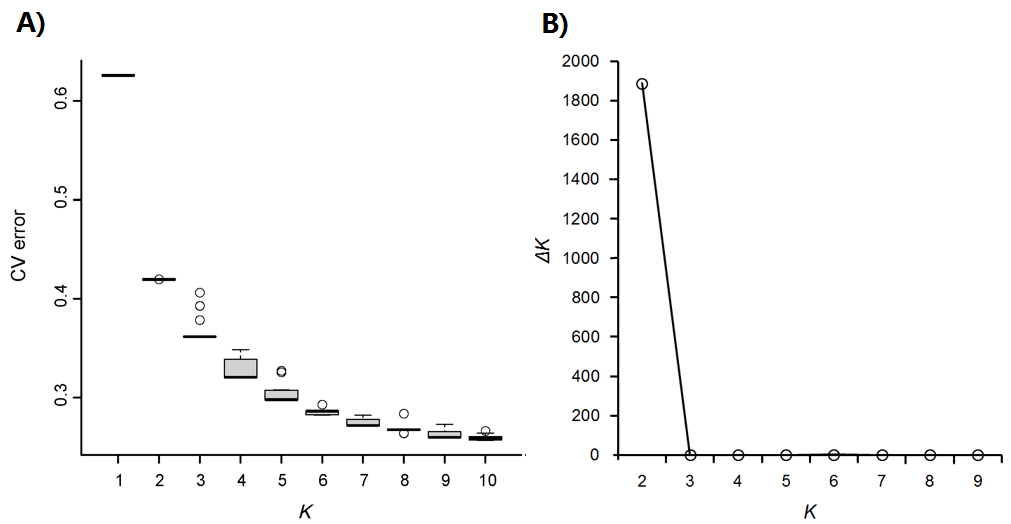

Supplement: dsac003_Supplementary_Data [file dsac003_supplementary_data.zip › Supplementary Figure 6.tif]
